# Barriers to leprosy elimination in Bolivia: Exploring perspectives and experiences of medical professionals and leprosy patients–A phenomenological study

**DOI:** 10.1371/journal.pntd.0013345

**Published:** 2025-08-11

**Authors:** Paula Messa Carmona, Nita Chaudhuri, Abundio Baptista Mora, Deisy Zurita Paniagua, Matthew Willis, Fabian Schlumberger, Nimer Ortuño-Gutiérrez, Anil Fastenau

**Affiliations:** 1 Department of Health Ethics and Society, Faculty of Health, Medicine and Life Sciences, Maastricht University, Maastricht, The Netherlands; 2 German Leprosy and Tuberculosis Relief Association (GLRA), Sucre, Bolivia; 3 Department of Global Health, Institute of Public Health and Nursing Research, University of Bremen, Bremen, Germany; 4 Marie Adelaide Leprosy Center (MALC), Karachi, Pakistan; 5 Damien Foundation, Brussels, Belgium; 6 German Leprosy and Tuberculosis Relief Association (GLRA), Wuerzburg, Germany; 7 Heidelberg Institute of Global Health, University of Heidelberg, Heidelberg, Germany; London School of Hygiene and Tropical Medicine, UNITED KINGDOM OF GREAT BRITAIN AND NORTHERN IRELAND

## Abstract

**Background:**

Leprosy elimination has recently re-entered the global health sphere, with the World Health Organisation’s (WHO) “Towards zero leprosy” strategy (2021–2030). Previously, its elimination had been defined as a prevalence of less than 1 case per 10,000, which was achieved on a global scale in 2000, leading to a large withdrawal of resources from leprosy control and to neglect on both global and national scales. Despite this, leprosy continued to spread and affect hundreds of thousands of people annually.

**Methods:**

The study explores the barriers to leprosy elimination in Bolivia, using a phenomenological approach, to discover the perceptions and experiences of leprosy patients and medical professionals regarding leprosy in Bolivia. It also explores the role of active case finding (ACF) for leprosy elimination in Bolivia. In-depth semi-structured interviews were conducted in Spanish, mainly at Jorochito Dermatological Hospital, the national referral centre for leprosy in Bolivia.

**Results:**

Barriers to leprosy elimination in Bolivia are present at provider, patient, governmental, societal and community levels. These include poor health financing, untrained workforce, poor treatment adherence, centralised organisation of leprosy diagnosis and treatment and health illiteracy.

**Conclusion:**

The barriers to leprosy elimination in Bolivia are complex, interconnected and embedded in Bolivian society. Leprosy elimination must be given priority on global and national scales to increase funding and importance, to continue ACF activities, and to promote national solutions for sustainable leprosy control.

## Author summary

Leprosy is a disease that has been eliminated in higher income countries but persists in lower income countries. This qualitative study explores the reasons that leprosy persists in Bolivia, by learning from the lived experiences of leprosy patients and medical professionals. 17 interviews were conducted in total; 12 with leprosy patients and 5 with medical professionals. The study also explored the role that Active Case Finding (ACF) plays in eliminating leprosy. ACF is a method of finding leprosy cases that can involve medical professionals examining high risk groups and searching for signs of leprosy. The study gave rise to a range of reasons that leprosy remains in Bolivia, termed “barriers to leprosy elimination” in the study, including (1) health system factors; (2) health service factors; (3) individual-related factors; (4) governmental neglect and NGO dependency; (5) weaknesses of the National Leprosy Programme and current leprosy control strategies; (6) societal stigma; and (7) lack of public knowledge. The study also found that ACF is an important strategy that must be prioritised to ensure that leprosy is eliminated in Bolivia.

## Introduction

### Background

Leprosy, also known as Hansen’s disease, is a neglected tropical disease (NTD), caused by the bacteria *Mycobacterium leprae* that causes skin lesions, affects peripheral nerve function, and can lead to disabilities if left untreated [1]. It is an ancient disease that persists in the modern world due to poverty and weak health systems [2]. Over 200,000 new cases are reported annually in over 120 countries, but many more cases remain unaccounted for globally. To successfully eliminate leprosy, the chain of transmission has to be interrupted [3]. Recent recommendations by the World Health Organisation (WHO) include active case finding (ACF) and preventative chemotherapy as measures to interrupt the chain of transmission [1]. This forms part of a larger strategy for leprosy elimination, the WHO “Towards zero leprosy” strategy (2021–2030) [3], which strives towards zero infection and disease, zero disability and zero stigma and discrimination.

**Fig 1:**
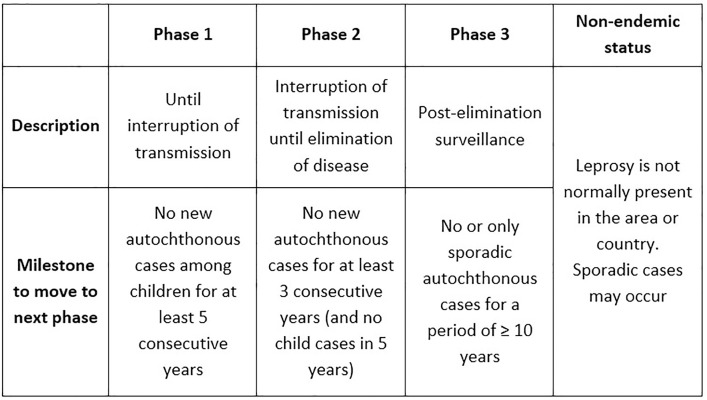
Leprosy elimination framework adapted from WHO [5].

For many years, leprosy has not been considered a public health threat, on a global or national level. This lack of impetus has recently been addressed by the WHO’s “Towards zero leprosy” strategy, which has redefined leprosy elimination, as “interrupting transmission and achieving zero autochthonous cases”, meaning zero cases of leprosy in a geographical country or area [3,4]. Additionally, the WHO published technical guidance on the “Interruption of transmission and elimination of leprosy disease”, consisting of a Leprosy Elimination Framework [5]. This framework consists of different phases of leprosy elimination and elaborates on the definition of leprosy elimination. It is Phase 2 that consists of the interruption of transmission until elimination of disease, which is measured by “no new autochthonous cases for at least 3 consecutive years (and no child cases in 5 years)” [5]. The full Leprosy Elimination Framework is shown in Fig 1.

The WHO Americas region reports the second highest new annual leprosy cases, with 29,936 in 2019. Brazil accounts for the majority of the leprosy burden in the WHO Americas region, but other Latin American countries, including Colombia, Paraguay, Venezuela, Argentina, and Bolivia continue to report cases annually [6].

In Bolivia, leprosy was endemic for centuries and it is now classified as a low-endemic setting for leprosy [2,7,8]. In 2006, there were 165 new annual cases reported in Bolivia [2]. This has been slowly decreasing, with 38 new cases reported in 2022 [2]. Despite the low incidence rate, children below 14 years of age make up 3% of the new annual cases in Bolivia, showing that active transmission of leprosy remains [7]. New cases among children means that Bolivia remains in Phase 1 of the WHO’s Leprosy Elimination Framework [5,9]. To progress to Phase 2, there must be no new cases among children for at least 5 consecutive years, as shown in Fig 1.

Within Bolivia, the region of Santa Cruz has historically reported the highest number of new leprosy cases annually (out of 9 regions), and continues to do so today [2]. The region of Santa Cruz shares a border with Brazil, which serves as a key leprosy entry point, as does the neighbouring region of Beni [2]. Jorochito Dermatological Hospital is located 43km outside the city of Santa Cruz. Since opening in 1972, it has been responsible for the diagnosis, treatment, and reporting of all leprosy cases within Santa Cruz, and is also the national referral hospital for leprosy cases in Bolivia [2]. Most leprosy diagnoses are made passively at Jorochito Dermatological Hospital, but 6 new cases were identified with ACF during household visits from 2021-2023, in a community-based intervention by Deutsche Lepra- und Tuberkulosehilfe e.V (DAHW) and the Damien Foundation [7,10].

This study explores the barriers to leprosy elimination in Bolivia from the perspective of Bolivian leprosy patients and medical professionals and in doing so aims to contribute to the research priorities of the WHO’s “Towards zero leprosy” roadmap. The main research question is *‘What are the barriers to leprosy elimination in Bolivia?’.* The study also addresses the sub question *‘What is the role of ACF for leprosy elimination in Bolivia?’*. By exploring the perspective of leprosy patients and medical professionals, current challenges, and insights into the Bolivian context, the study aims to provide recommendations that can contribute to the elimination of leprosy in Bolivia. These recommendations are relevant for the national government, the regional government of Santa Cruz, as well as for non-governmental organisations (NGOs) working to reduce leprosy in Bolivia, including the Damien Foundation and the DAHW [11,12].

## Methods

### Ethics statement

The study has been approved by the Maastricht University Programme Ethical Review Committee, with the registration number FHML/GH_2024.01. Ethical clearance in Bolivia was also obtained and approved by the ethical review board of the Universidad Mayor Real y Pontificia de San Francisco Xavier of Chuquisaca. Informed consent was gained from all participants using the consent form attached as *appendix 1.*

### Study design

A phenomenological approach was used to address the research questions. This qualitative approach allowed for the investigation of the barriers to leprosy elimination from the lived experiences of the participants [13]. Semi-structured in-depth interviews were conducted, in line with the phenomenological approach. Semi-structured interviews provide flexibility to validate participants’ answers, resulting in the collection of accurate and reliable data [14].

### Sampling method

Purposive sampling was used for the recruitment of patients and medical professionals with first-hand experience of leprosy in Bolivia [15]. This was necessary as the aims of the study define the specific types of participants required to answer the main research questions [15,16]. Recruitment took place from mid-May to mid-June 2024. The inclusion and exclusion criteria for participation in the study is outlined in Table 1.

**Table 1:** Inclusion and exclusion criteria for participation.

	Inclusion	Exclusion
**Leprosy patients**	Aged 18 or over Confirmed leprosy diagnosis Undergoing leprosy treatment or completed Ability to speak Spanish	Unable to give informed consent Considered to be too unwell to participate by doctors
**Medical Professionals**	Aged 18 or over Ability to speak Spanish Medical professionals with experience with leprosy patients/ control	Unable to give informed consent

### Study setting

The data collection was carried out in the region of Santa Cruz, the largest region in Bolivia by population and area, principally at Jorochito Dermatological Hospital. Some interviews were conducted at patients’ houses during ACF household visits, and one in the city of Santa Cruz with a medical professional.

### Research population

There was a total of 17 participants, including 12 leprosy patients and five medical professionals. Demographic data of the participants are outlined in Tables 2 and 3. This was dependent on the availability and willingness of leprosy patients and medical professionals at Jorochito Dermatological Hospital as well as during ACF household visits. The medical professionals were all medical doctors with experience in diagnosing and treating leprosy patients, and 3 of them had additional experience in other leprosy control activities, including ACF and involvement in the National Leprosy Programme (NLP) in Bolivia. The interviewed leprosy patients had differing forms of leprosy (PB or MB), different disability grades as per the WHO grading system, and some had leprosy reactions [17].

**Table 2:** Participant information: Leprosy patients.

Variables		Number of participants (total = 12)		
**Sex**	Male	7		
	Female	5		
**Age**	18-25	1		
	26-35	4		
	36-45	1		
	46-55	3		
	66-74	1		
	75+	2		
**Leprosy type**	PB	1		
	MB	11		
**Leprosy reaction**	Yes	4		
	No	8		
**WHO disability grading**		***G0D***	***G1D***	***G2D***
	Hands	9	3	0
	Feet	7	5	0
	Eyes	10	1	1

**Table 3:** Participant information: Medical professionals.

Variables		Number of participants (total = 5)
**Sex**	Male	3
	Female	2
**Profession**	Dermatologist	2
	Medical coordinator Damien Foundation	2
	Ex-director NLP Bolivia	1

### Data collection procedures

The data collection took place over one month, beginning in mid-May 2024. In-depth semi-structured interviews were conducted in Spanish. First, the potential participants were informed of the study and given the opportunity to ask any questions. Then, potential participants were asked if they wanted to participate in the study and invited to sign a consent form - *appendix 1*. Once the consent form was signed, the interview was conducted and recorded for analysis purposes. The interviews were conducted using a pre-prepared interview guide. Separate interview guides were prepared for the leprosy patients and medical professionals, consisting of 6 guiding questions, to allow the participants to explain their experience with leprosy attached as *appendix 2* and *3* respectively. The interviews on average lasted 16:40 minutes, ranging from 4:52 minutes to 55:43 minutes. Medical professional 5 provided electronic, written answers to the questions instead of conducting an in-person interview due to personal preference.

### Data analysis

The interview recordings were manually transcribed verbatim by PMC. The transcribed text was manually translated from Spanish to English by PMC. To begin with, the translation was read and re-read to increase familiarity with the data. A thematic analysis was conducted, using ATLAS.ti to assist with coding the interviews into themes. Both deductive and inductive approaches were conducted. In this way, the analysis was guided by pre-established themes grounded in the literature, but also gave rise to new, emerging themes relevant to leprosy elimination in Bolivia [18].

## Results

Themes grounded in the literature arose during the analysis, including: (1) health system factors; (2) health service factors; and (3) individual-related factors. Additional themes emerged inductively during the analysis, including: (4) governmental neglect and NGO dependency; (5) weaknesses of NLP and current leprosy control strategies; (6) societal stigma; and (7) lack of public knowledge.

### Health system factors

Factors related to the health system were identified as barriers to leprosy elimination, namely health workforce and health financing.

#### Health workforce.

Several medical professionals reported a lack of knowledge of leprosy within the workforce. It was reported that some dermatologists cannot distinguish leprosy lesions against lesions caused by other diseases and therefore can misdiagnose leprosy patients.

Due to the lack of knowledge and consequent misdiagnoses, various patients reported that they had to visit several medical professionals before leprosy was considered a cause for their lesions. These misdiagnoses and consequent delay in diagnosis is a significant barrier to leprosy elimination. Without a timely diagnosis and commencement of treatment, leprosy transmission continues, preventing its elimination.

This lack of knowledge and resultant misdiagnoses attributed to a lack of training at university/degree level, and a lack of additional training or professional development.

*“The health services don’t have the capacity to deal with leprosy… They just don’t know. If you don’t know how you are going to be able to do that. Right? So, this is one of the weaknesses that I have found. The health services are not trained at all” – Medical Professional 2*

Additionally, lack of knowledge was associated with fear in the health workforce towards leprosy patients and the disease. Medical Professional 2 reported that leprosy trainings delivered to health workforce in the past had not been received in the intended manner due to fear.

*“One of the main problems is that we don’t have the response from the health personnel that we would like to have. When you go to teach them and tell them to prioritise the issue of leprosy. Why? Because there is fear. The health personnel themselves are afraid. Because when we tell them… there is a leprosy patient over there and you are going to monitor him. It turns out that this leprosy patient has not been cured or monitored even once” – Medical Professional 2*

Frequent staff turnover within the health workforce was also reported to impact knowledge about leprosy. The impact of training courses, designed and delivered to build on existing knowledge, is lessened as changes in staff prevent workers from participating in follow-up sessions.

Leprosy patients reported instances of stigma and discrimination by health workforce while seeking treatment for their lesions. This is demonstrated by an incident reported by Patient 4.

*“Which is why I didn’t even go to hospitals because when I had my wound…The nurses didn’t want to clean me or, well, they would clean me and when I showed them what illness it was, they would grab me and write me a note telling me to buy gloves” – Patient 4*

Stigma against leprosy patients was also reported by the medical professionals.

*“And sometimes they don’t even cure them, because there is always that fear, that stigma against leprosy.” – Medical Professional 4.*

There is also a lack of leprosy specialists working in the diagnosis and treatment of the disease in Bolivia. In the department of Santa Cruz, there are just two leprosy specialists carrying out ACF and other leprosy control activities, with support from the Damien Foundation.

#### Health financing.

Despite the free health services across Bolivia, poor financing mechanisms result in low-quality care. This is a significant barrier to leprosy elimination, as the majority of leprosy patients in Bolivia are of low-socioeconomic status and rely on free services [2]. The poor quality of services results in frequent leprosy misdiagnoses, and patients may spend years seeking correct treatment. Due to the poor attention and misdiagnoses received at public health services, people often turn to private healthcare, which is often unaffordable. Patient 5 expressed a desire to cure their leprosy-related lesions and ulcers at a private clinic but were unable to due to high costs. Additionally, Jorochito Dermatological Hospital is also affected by the lack of financing, despite being the national referral hospital for leprosy.

### Health service factors

Factors related to health service were identified as barriers, including availability, affordability, appropriateness and treatment (availability, adverse reactions, adherence).

#### Availability.

Availability is defined as the physical existence of health resources with the capacity to produce services [19,20]. Both leprosy patients and medical professionals reported difficulties in accessing Jorochito Dermatological Hospital, often having to travel far to reach it. The distance to Jorochito Dermatological Hospital limits access to diagnosis, initial treatment and ongoing care. Patients are required to return monthly for check-ups and to collect their medication; a challenge that often leads to abandonment of treatment. Medical professionals reported that the lack of decentralised services to diagnose and treat leprosy is a key barrier to its elimination. A decentralised approach to leprosy control would allow for more equitable access to diagnosis, treatment and care.

#### Affordability.

Affordability is defined as the economic ability to spend resources and time to access the necessary services, including additional related costs [19,20]. Several patients and medical professionals reported that limited resources and financial constraints prevent leprosy patients from accessing and adhering to treatment at Jorochito Dermatological Hospital. This is a significant barrier to leprosy elimination, especially as leprosy is described as a disease of poverty, often found in people with a low socioeconomic status [2].

*“I’m unemployed, have little resources, so I couldn’t come here to the hospital to see if they could cure me.” – Patient 3*

Patients sometimes turn to traditional medicine to be treated, due to its affordability and availability. Traditional medicine is not a viable treatment for leprosy; patients’ lesions can worsen, transmission continues, and disabilities can develop, ultimately further hindering progress towards leprosy elimination.

#### Appropriateness.

Appropriateness, defined as the fit between patients’ needs and the health service, as well as its quality [19,20], was found as patients reported experiences with inappropriate health services, where their leprosy was not successfully diagnosed or treated.

*“They gave me creams and injections and everything, but it didn’t have any effect.” – Patient 1*

When patients are prescribed incorrect medication, leprosy progresses to more debilitating stages. A delay in diagnosis is problematic for leprosy elimination, as the infected person remains contagious and can lead to an increase in leprosy cases. One of the patients recognised that while they were searching for treatment, their skin lesions worsened. Another reported that at one of the health services they had visited, the doctors had wanted to amputate their legs.

#### Treatment (Availability, adverse reactions, adherence).

Barriers were identified in relation to treatment availability, adverse reactions, and adherence. It was reported that only Jorochito Dermatological Hospital administers the free leprosy treatment provided by the WHO in Bolivia. The centralised nature hinders treatment adherence, due to related distance and costs.

Additional barriers to adherence were identified. A patient reported that the side effects of the treatment made them feel too unwell to continue taking it. The side effects of the treatment and consequent abandonment is a barrier to leprosy elimination on a global scale, as completing the treatment regime is extremely important to ensure that leprosy patients are no longer contagious.

*“[I had] three tablets or four tablets left over because I couldn’t do it anymore… It made me vomit and by that time I was smelling blood, and I was going to the bathroom. So that’s why I stopped.” – Patient 9*

Poor treatment adherence was also reported due to stigma and the social environment in Bolivia. Medical professionals reported that leprosy patients are less likely to go for check-ups and complete their treatment regime compared to tuberculosis patients, due to stigma.

*“I have been able to consult many people, many people. Why haven’t you gone to your check-up? No, because of the pandemic, because of the pandemic, I was already scared. And how, how did your brother or your neighbour who has tuberculosis go to his check-ups? What happens is that leprosy has a very important component of stigma, of stigma, that they take advantage of any circumstance not to, not to be there, not to expose their illness in front of everyone.” – Medical professional 2*

*“The cultural aspect that affects their compliance with their treatment scheme.” – Medical Professional 5, written answer*

### Individual-related factors

Factors related to the individual refer to the leprosy patients’ knowledge, perception, health literacy, and stigma.

#### Knowledge.

It was reported that some patients were not aware that leprosy continues to exist in today’s society at the time of their diagnosis. This lack of knowledge, reported by both patients and medical professionals, is a significant barrier to elimination, as it results in patients receiving a delayed diagnosis, due to a lack of urgency in seeking/starting treatment.

Another patient reported knowing what leprosy was, but described it as being characterised by rotting flesh, and consequently not believing they had leprosy. This lack of knowledge and denial about leprosy was also reported by medical professionals. Patients in denial about their diagnosis are more likely to abandon treatment, and as denial is also closely interlinked with stigma, it remains a significant barrier to elimination.

#### Perception.

Another barrier to leprosy elimination is patients’ perceptions towards leprosy and its treatment. It was reported that patients prefer injections over tablets, as they believe injections result in fewer side effects. This perception of tablets and injections was reported by both patients and medical professionals and can hinder treatment adherence.

*“I wish it wasn’t so many medications, I wish it was just a few injections, because over time… medications are bad for the liver…I’ve had issues with my gall bladder and I’m afraid that all that will affect me again. That’s why I often refuse to take a lot of medication.” – Patient 4*

Medical professionals reported that the perception of sensitivity and pain is a barrier to elimination, as diseases are associated with pain and discomfort, so patients often do not understand that a lack of sensitivity can indicate a serious disease. Additionally, past experiences with skin pathologies mean that patients rely on familiar treatments that they believe to be effective but are not for the treatment of leprosy. This is a barrier, as it delays receiving the correct diagnosis and commencing the necessary treatment to manage leprosy.

#### Health literacy.

Poor health literacy was identified, as marks on the skin are not considered a priority or are regarded as something that patients have dealt with before. As a result, symptoms are not communicated to health professionals, and a timely leprosy diagnosis cannot be made.

In addition, other leprosy patients reported that they did not consider their initial symptoms to be serious and were therefore ignored, resulting in a delayed diagnosis and access to the treatment.

*“First I got a few small marks… on my arm, and I didn’t think much of it.” – Patient 7*

Poor health literacy also manifests itself in a lack of understanding of the disease and treatment. As a result, leprosy patients miss their monthly check-ups and do not adhere strictly to the treatment, and therefore remain an active leprosy case prone to furthering transmission.

#### Stigma.

Leprosy patients can have stigma about their condition and refuse to accept it as their reality, hindering treatment adherence and resulting in leprosy patients abandoning treatment and seeking alternative options.

Stigma at the patient level also manifests itself in fear, hiding, and isolation. This is likely to cause a delay in seeking and receiving the correct diagnosis and treatment, resulting in an increased possibility of transmission.

*“We have found lepers in the market, serving people, shaking hands with whoever. But the moment they find out that they are a leper, they immediately ran away. They don’t know what leprosy is, that’s the truth.” – Medical Professional 3*

Stigma at the patient level can prevent ACF. Instances of leprosy patients refusing visits to their homes were reported – they do not share their address with the ACF team or prefer to meet in a different location. This adds a level to complexity to ACF as the best setting for it is the home, where one can have personal conversations, the doctors can share information with the patient, and family members are present to take the single-dose of rifampicin post exposure-prophylaxis (SDR-PEP).

### Governmental neglect and NGO dependency

Neglect by the Bolivian government was reported by both patients and medical professionals. The lack of priority given to healthcare is reflected by the weak health system that cannot provide the necessary primary health care, let alone detect and treat leprosy in a timely manner. Not only does the government neglect health as a whole, but leprosy as a disease is specifically neglected. While the neglect of leprosy can be seen on a global scale and by other national authorities, it remains a significant barrier to the elimination of leprosy in Bolivia.

*“Leprosy is a neglected disease, so the government does not budget anything for leprosy.” – Medical professional 4*

As a result of the neglect of healthcare and of leprosy in Bolivia, external actors play a prominent role to supplement the actions of the government. For leprosy, NGOs are funding and organising the control strategies. This inaction at the governmental level is a significant barrier to leprosy elimination, as it results in a lack of national policies, action plans, structures and personnel, that are all required for leprosy elimination.

However, problems with the role of NGOs in leprosy control were also identified. Firstly, the lack of importance given to leprosy on a global scale results in a lack of funding. This lack of funding means that NGOs do not have the means to support leprosy control in Bolivia, and those that can provide support have a very limited budget, meaning the control strategies are also limited. Secondly, medical professionals reported biases and challenges that arise from the work of NGOs. These challenges with NGOs, despite them being the only current source of funding for leprosy control activities in Bolivia, show that their presence is a temporary solution, and the ultimate aim is that the government gives importance and funding to leprosy control.

An alternative perspective to leprosy control was also reported. Medical professional 3 described that leprosy control has to be achieved in a sustainable manner, through teaching others and implementing strategies that work for Bolivia.

*“If we are going to help, let’s do it by making people take responsibility with what they have. Let them do well with what they have… Not by bringing me help from outside, but with what I have to teach me and so that I can do it.” – Medical professional 3*

### Weaknesses of National Leprosy Programme and current leprosy control strategies

It was reported that the NLP in Bolivia consists solely of the arrival of the medication from the WHO. Additionally, there is a “*lack of a leprosy control task force*” (Medical professional 5, written answer), further hindering the possibility of an effective NLP. It was reported that the national leprosy control strategies are just being carried out at Jorochito Dermatological Hospital with the support from Damien Foundation.

Challenges with the current leprosy control activities were also reported, including the lack of follow-up mechanism and the lack of facilities that can diagnose and treat leprosy. These reports demonstrate how the current leprosy strategies are insufficient to diagnose and treat all leprosy cases in Bolivia and in preventing new cases.

### Societal stigma

Societal stigma was identified in addition to the stigma at the individual patient level. It was reported that stigma is found in several aspects of Bolivian society, hindering leprosy control efforts and posing a barrier to leprosy elimination. Stigma leads to rejection of patients by their families and employers, causing a simultaneous loss of a support system and an income. This rejection causes leprosy patients to be isolated and hinders treatment adherence.

“*Well, the simple fact is that as long as you say leprosy, people tend to reject you.” – Patient 8*

Stigma from the society also projects itself onto the health system. Medical professional 4 reported that *“if I put dermatologist, leprosy specialist, in my office, nobody goes to my office”.* This is problematic, as it shows that medical professionals also have to hide the fact they are associated with leprosy. This sense of hiding and secrecy only make diagnosing and treating leprosy in a timely manner more challenging. Stigma was also identified from other authoritative figures, including teachers and the church, demonstrating how ingrained stigma is in Bolivian society.

Stigma was also reported due to the mythical connotations surrounding leprosy. It results in misinformation about the spread of leprosy and rejection of leprosy patients, posing barriers to leprosy elimination.

*“It has been a little bit that people still have that old myth that it is contagious just by touching it. So yes, we have noticed that rejection, but not from the family, but from the society.” – Patient 10*

Stigma means that anything leprosy related will not be well attended. This could include training for medical professionals, free skin clinics for communities where known active patients reside, or a workshop to inform the general public. It was reported that a mobile skin clinic, intended to detect leprosy, had to be advertised as a clinic for any skin pathology due to the risk of poor attendance. Not only does this affect the efficiency of skin clinics to detect leprosy cases, but it also demonstrates the challenges of addressing leprosy on a large scale in Bolivia.

### Lack of public knowledge

There were several reports about a lack of knowledge about leprosy, specifically regarding the prevalence in Bolivia and its impact on public health. This lack of knowledge results in a delayed diagnosis, as people are unaware that their lesions may be leprosy. The lack of knowledge about the role of leprosy in today’s society also contributes to the stigma, due to the established mythical connotations and consequent fear, exclusion, and rejection.

*“For some people leprosy no longer exists, when we talk to them in the clinic and explain or comment on some occasions, they say ‘ah I thought it didn’t exist anymore’.” – Medical professional 1*

Lack of knowledge remains due to the poor health communication regarding leprosy to the general public. In order for leprosy to be eliminated, the general public must be well informed about the signs and symptoms, how to access the necessary care, and the importance of treatment adherence to prevent the spread to others.

### The role of active case finding for leprosy elimination

The sub-question of this study is to investigate the role of ACF for leprosy elimination in Bolivia. Firstly, medical professionals described ACF as “*important*” (Medical professional 4 & 5) and as the strategy that “*deserves all, all, all, all, all the priority for diagnosis*” (Medical professional 2). There are several reasons for its importance for leprosy elimination, including early case detection within close contacts of active patients and administration of SDR-PEP to contacts to prevent the development of leprosy.

*“The active search for leprosy cases is very important in order to find cases early and treat them in a shorter period of time, thus preventing many transmissions and avoiding disabilities. Post-exposure prophylaxis is very important to prevent the development of the disease in their contacts.” – Medical professional 5, written answer*

Another reason that ACF is so important is because of the clear exposure to leprosy faced by household contacts of leprosy patients. The household of an active leprosy patient is a likely place of transmission, and therefore the household contacts must be visited to check for signs of leprosy, and if no signs are present then SDR-PEP must be taken in case leprosy is in its initial, hidden stages.

*“The active search is basic, elementary, because one will be able to enter the person’s home where they live, eat, share with their intimate ones. So, there is no other place. There is no other place where people with a higher risk are, such as contacts with an index case.” – Medical professional 2*

The medical professionals also described how ACF should be performed, as there have been instances in the past where ACF has resulted in incorrect diagnosis of leprosy, which has personal consequences for those misdiagnosed. ACF must be conducted by trained medical professionals among the close contacts of an active leprosy case. This way the search is refined, and it is known that there has been close contact with a leprosy patient.

ACF also gives medical professionals the opportunity to educate and de-mystify the disease. As discussed above, this is not something that can be done on a large scale due to stigma, but in the household, it is easier, and patients are more receptive to learning.

*“At home is where all this has to be done. It is the place where you can teach them. Where you can demystify. To tell them what the healer or the grandmother or the aunt has told them, that this is an illness related to the mythical, religious or telluric part, or that someone has cursed you.” – Medical professional 2*

It was also reported ACF is especially relevant in Bolivia, due to the low number of leprosy cases. With the necessary funding and organisation, it is possible to conduct ACF effectively and visit every active leprosy case in Bolivia.

*“Above all, we are in a country where the burden of leprosy is low. So, there is no reason for us to not go there. There is no reason.” – Medical professional 2*

It is clear that ACF plays an indispensable role for leprosy control in Bolivia and is an essential part of the strategy for leprosy elimination. It must be prioritised as a key strategy to prevent the development of new cases and to diagnose new cases in a timely manner to prevent transmission to others.

## Discussion

The barriers to leprosy elimination in Bolivia span provider, patient, societal, community, and governmental levels. Firstly, provider level factors encompasses both health system factors (e.g., health workforce and financing), and health service factors, (e.g., availability, appropriateness and affordability of the health service).

Within the health workforce, a lack of knowledge about leprosy causes misdiagnoses and delayed diagnoses. A recent qualitative study found this stems from both inadequate training at the university level and a lack of professional development [7]. Moreover, Bolivia has only two leprologists conducting ACF and other control activities, which, combined with limited funding, presents a major challenge to leprosy elimination. This leads onto the next identified barrier to leprosy elimination; health financing, or the lack thereof. Limited public healthcare funding hinders timely diagnosis and treatment, contributing to poor treatment adherence and continued transmission. In Bolivia, only Jorochito Dermatological Hospital diagnoses and treats leprosy, yet due to minimal government support it must rely on patient fees to run.

Next, barriers concerning the health service were found to hinder leprosy elimination. The limited availability of health services in Bolivia hinders access to leprosy care. Patients often travel long distances to the sole treatment centre, making the centralised nature of care a significant barrier. Decentralising services has been shown to reduce diagnostic delays and disability cases [21]. The unaffordability of health services, which includes all the costs associated with accessing care, limits treatment adherence and can result in patients seeking cheaper, more accessible treatment such as traditional medicine. Furthermore, inappropriateness of the health service, where care does not align with patient needs, often results in underdiagnoses and misdiagnoses of leprosy. This leads to progression of the disease and an increased chance of transmission to others. Access and adherence to leprosy treatment also presents challenges. Leprosy treatment can only be accessed at Jorochito Dermatological Hospital and patients have to go once a month to collect it throughout their treatment. Patients that live far away, do not have the resources to pay for transport, or cannot take time off work, often end up abandoning the treatment. Side effects of the medication, as well as stigma, also drive patients to abandon treatment, hampering elimination efforts. Lack of treatment adherence is a major barrier for leprosy elimination and should be the focus of future interventions to reduce the leprosy burden. Interventions such as cash transfer and tele-health programs have been shown to improve adherence, and could therefore be implemented in Bolivia to contribute to leprosy elimination [22,23].

Patient level factors were also identified, which include knowledge, perception, and health literacy. Many patients’ lack awareness of leprosy symptoms and its continued endemicity in Bolivia, leading to denial of the diagnosis and consequent treatment abandonment. This lack of knowledge impedes early detection and ACF, and contributes to the existing stigma [24]. Moreover, patients’ perceptions of leprosy hinder care. Some view it as a skin condition treatable by natural remedies, while others’ preference towards injections makes adherence to oral treatment more challenging. Similarly, health illiteracy is a barrier, with skin pathologies not considered important or as requiring urgent medical attention, leading to a lack of awareness about the need to strictly adhere to and complete the treatment. Poor health literacy is also strongly intertwined with stigma; interventions that improve health literacy have been shown to reduce internalised stigma [25,26]. Stigma at the patient level fosters isolation and fear, preventing access to health services and encouraging treatment abandonment [27]. Stigma can also hinder ACF activities, for example if patients refuse to be visited in their homes or decline their household contacts to be screened for fear of stigmatisation.

Additional barriers to leprosy elimination include barriers at the societal and governmental level, namely governmental neglect of the health system and of the NLP. National governments play a critical role in resource mobilisation and strategic partnerships for disease elimination [3,28]. Political apathy in Bolivia towards leprosy has resulted in an NLP that merely organises the arrival of WHO supplied leprosy treatment. With no leprosy control task force, national policies, or strategies in place, the prospect of leprosy elimination seems distant. Leprosy control initiatives currently rely heavily on Damien Foundation funding due to the absence of comprehensive government backing. Moreover, the lack of centralised clinical management and monitoring systems hinder effective surveillance. Effective surveillance is crucial in low-incidence settings to ensure timely diagnosis and treatment [3]. Moreover, stigma at the societal level was identified as a barrier. Rejection by family, friends, employers, teachers, or the church is extremely damaging for leprosy patients; it has even been described as the most powerful barrier to leprosy elimination [29]. Stigma rooted in historical and mythical connotations is embedded in Bolivian society and will require a collaborative effort to overcome [30]. Unfortunately, stigma is also present in health services and perpetuated by some medical professionals. This was also reported by a recent qualitative study that explored knowledge, attitudes, and practices of health staff in Bolivia [7]. This stigma further hinders early case detection, treatment adherence and appropriate clinical management for leprosy. Finally, a lack of public knowledge about leprosy was identified as a barrier; if rectified this could assist in early case detection and treatment adherence [31]. It would also increase the involvement of communities in leprosy control, which is essential for leprosy elimination [3,31,32].

### The role of active case finding for leprosy elimination in Bolivia

ACF involves proactive outreach to detect and treat leprosy cases, rather than waiting for patients to seek care [33]. In Bolivia, ACF consists of household visits to screen contacts and administer SDR-PEP if there are no clinical signs of leprosy. The importance of ACF for leprosy elimination was highlighted by the interviewed medical professionals, as it ensures that those individuals most susceptible to leprosy, i.e., those living in close contact with a leprosy patient, are evaluated, protected by SDR-PEP, or diagnosed and treated if appropriate. These findings are strongly supported by the literature, as ACF has been shown to find many patients in the early stages of leprosy reducing progression, disability, and transmission [34,35].

Bolivia’s current ACF strategy can be described as household contact tracing. Other forms of contact tracing, not yet implemented in Bolivia, include tracing social contacts, such as neighbourhood and school contacts [36]. There are also other methods of ACF, including door-to-door detection, rapid village screening, and the distribution of screening questionnaires [36]. A recent study explored the different types of ACF used for leprosy in high endemic regions vs in low endemic regions, and how effective the methods were in early case detection [36]. In low endemic settings, rapid village screening was found to be the most effective method in identifying new cases, i.e., greatest % of case detection. However, door-to-door detection and screening questionnaires were used to screen and evaluate the greatest number of individuals, compared to household contact tracing and rapid village screening. The screening of a large number of individuals is essential for leprosy elimination, specifically in low endemic regions like Bolivia [36]. To successfully eliminate leprosy in Bolivia, ACF strategies could be expanded to include a more generalised search, in combination with the targeted household visits. In this way, more individuals could be screened, increasing the chance of finding leprosy cases, providing treatment, and preventing transmission to others. This requires funding, organisation, trained personnel, and the structures in place to conduct the search, diagnose, treat, and monitor the new and existing leprosy patients.

ACF is integral to leprosy elimination efforts, highlighted by the WHO in the “Towards zero leprosy” strategy [3]. By directly reaching individuals and evaluating them, ACF overcomes several of the barriers that leprosy patients face at the beginning of their disease, in terms of accessing appropriate health services and obtaining a correct diagnosis. It also overcomes individual-related barriers, such as health illiteracy, perception, and internalised stigma, as ACF removes the responsibility from the patient to search for and obtain a correct diagnosis. ACF also gives medical professionals an opportunity to communicate, educate, and inform leprosy patients and their contacts about the disease, the treatment, and the importance of its strict adherence, and therefore increase awareness and prevent further transmission of the disease.

### Limitations

The study has methodological limitations that must be acknowledged. To begin with, the study was conducted in the region of Santa Cruz, and therefore is not generalisable to the whole of Bolivia. This setting was selected as it is where Jorochito Dermatological Hospital, the national referral hospital for leprosy, is located. Santa Cruz is also one of the two regions supported by the Damien Foundation, and therefore where several leprosy control activities are being carried out in Bolivia. Additionally, the small number of participants, and the fact that only one participant had grade 2 disability in their eyes, may have resulted in certain perspectives and experiences being missed. Furthermore, there are limitations associated with using interviews as the sole data collection method [37]. Interviews are unable to capture the full, true picture, due to the artificial setting, lack of familiarity between the interviewer and interviewee, and perhaps also due to stigma. In this study, a small number of participants were interviewed, which is therefore not representative of all the barriers to leprosy elimination in Bolivia. The interviewed medical professionals either worked at Jorochito Dermatological Hospital or were connections of the Damien Foundation, and therefore contain a degree of selection bias. There are also limitations regarding the data analysis process. During the deductive coding, several of the themes and sub-themes from the literature were overlapping, making it difficult to assign themes to codes. Additionally, as this is a subjective process, it may not be fully naturalistic, that is to fully capture the participants’ experiences in the findings [38]. Inductive coding was also conducted, which again is a subjective process and therefore may not be fully naturalistic.

### Recommendations

**Fig 2:**
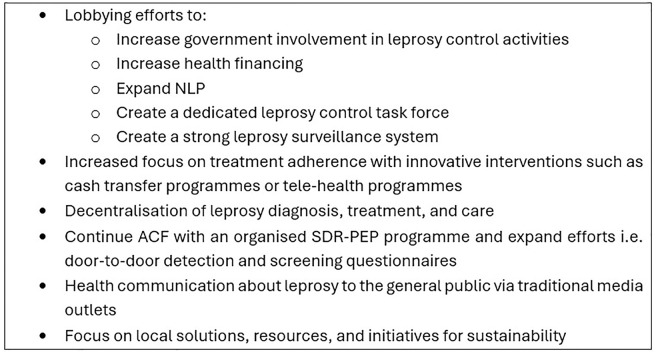
Summary of recommendations for leprosy elimination.

Recommendations to reduce the leprosy burden and contribute to leprosy elimination in Bolivia have been formed based on the findings. The recommendations, summarised in Fig 2, require funding and a large collaborative effort, and are in alignment with the WHO “Towards zero leprosy” strategy [3].

## Conclusion

In conclusion, the study has described several barriers to leprosy elimination in Bolivia, has explored the crucial role of ACF for leprosy elimination and has provided recommendations to contribute to current leprosy elimination efforts in Bolivia. It is clear that the described barriers are complex, highly interconnected, and embedded in Bolivian society. There is no quick solution for the described barriers to leprosy elimination; it requires time, effort, and funding. It requires collaboration between the Bolivian government (national, regional and local), the Damien Foundation, actors across several health departments, e.g., public health, epidemiology, primary health care etc., medical professionals, leprosy patients, and many more actors. It requires health system strengthening, social development, and support for marginalised communities and individuals. It requires a focus on stigma reduction at a societal level, within the health system and on an individual, patient level. It requires continued focus on ACF for early case detection and to monitor the most susceptible individuals (household contacts of a leprosy patient). It requires a focus on treatment adherence, to ensure leprosy patients are treated and can no longer transmit the disease to others. Leprosy and its elimination must be given importance and priority, not just within Bolivia but also on a global scale, in order for leprosy elimination to become a reality.

## Supporting information

S1 File Consent forms. (DOCX)

S2 File Interview guides for leprosy patients. (DOCX)

S3 File Interview guides for medical professionals. (DOCX)

S4 File Participant information sheets. (DOCX)

S1 Data Alternative Language Abstract. (DOCX)
