# Supplementary material for: Barriers to leprosy elimination in Bolivia: Exploring perspectives and experiences of medical professionals and leprosy patients–A phenomenological study
Source: PLoS Negl Trop Dis. 2025 Aug 11;19(8):e0013345. doi: 10.1371/journal.pntd.0013345 (PMC12338824; doi:10.1371/journal.pntd.0013345)
Supplement: S1 File — (DOCX) [file pntd.0013345.s001.docx]

**Appendix 1 - Consent forms, Spanish and English**

Formulario de consentimiento de participación

Quiero participar voluntariamente en un proyecto de investigación dirigido por Paula Messa-Carmona para el estudio «Barreras a la eliminación de la lepra en Bolivia», para completar su MSc Global Health en la Universidad de Maastricht, Países Bajos, y para su publicación con la Asociación Alemana de Ayuda contra la Lepra (DAHW) en una publicación científica.

Entiendo que:

|  | **Si** | **No** |  |
| --- | --- | --- | --- |
| 1. Mi participación en este estudio es voluntaria. No se me pagará por mi participación, y puedo retirarme y dejar de participar en cualquier momento sin penalización. | □ | □ |  |
| 1. Tengo derecho a no responder a cualquier pregunta. | □ | □ |  |
| 1. Puedo retirar mi participación en este estudio en cualquier momento sin dar razones y hacer que se borren mis datos. Entiendo que, una vez finalizado el análisis de la investigación, mis contribuciones no podrán extraerse y seguirán formando parte de la publicación. | □ | □ |  |
| 1. El estudio está diseñado únicamente para recopilar información para el estudio mencionado anteriormente para la realización del máster en Salud Global de la Universidad de Maastricht, y para su publicación en una publicación científica con DAHW. | □ | □ |  |
| 1. El investigador no me identificará por mi nombre en ninguna publicación que utilice información obtenida de este estudio, y mi confidencialidad como participante en este estudio permanecerá segura. | □ | □ |  |
| 1. Los datos recogidos para este estudio pasan a ser propiedad de la Universidad de Maastricht y no se compartirán con entidades externas. La información personal recopilada sobre mí que pueda identificarme, como mi nombre o edad, no se compartirá más allá de la investigadora principal. | □ | □ |  |
| 1. Los datos (transcripciones y grabaciones) se guardarán con precaución y durante 10 años después de su publicación, de acuerdo con el Código de Conducta de Gestión de Datos de Investigación de la Universidad de Maastricht. | □ | □ |  |
| 1. Los datos se guardarán en el ordenador portátil seguro de la investigadora durante el periodo de la tesis (Mayo- Agosto 2024) y en las instalaciones de almacenamiento de datos de la Universidad de Maastricht posteriormente. Entiendo que este es un lugar seguro. | □ | □ |  |
| Doy mi completo consentimiento para participar en este estudio.  **Firmas**  **Participante**  _____________ _____________________ ________  Nombre del participante Firma Fecha y lugar |  |  |  |
|  |  |  |  |

**Investigador**

Hice todo lo posible para asegurarme de que el participante entendiera a qué estaba dando su consentimiento voluntariamente, di al participante la oportunidad de hacer preguntas y confirmo que el participante ha dado su consentimiento libremente.

________________________ __________________ ________

Nombre del investigador Firma Fecha y lugar

Participation consent form

I volunteer to participate in a research project conducted by Paula Messa-Carmona for the study ‘Barriers to leprosy elimination in Bolivia’, for the completion of her MSc Global Health at Maastricht University, The Netherlands and for publication with the German Leprosy Relief Association (DAHW) in a scientific journal.

I understand that:

|  | **Yes** | **No** |  |
| --- | --- | --- | --- |
| 1. My participation in this study is voluntary. I will not be paid for my participation, and I may withdraw and discontinue participation at any time without penalty. | □ | □ |  |
| 1. I have the right to decline to answer any question. | □ | □ |  |
| 1. I can withdraw my participation in this study at any time without providing any reasons and have my data deleted. I understand that after the researcher’s analysis is complete, my inputs cannot be extracted and will remain a part of the publication. | □ | □ |  |
| 1. The study is designed to only gather information for the above-mentioned study for the completion of the masters in Global Health at Maastricht University, and for publication in a scientific journal with DAHW. | □ | □ |  |
| 1. The researcher will not identify me by name in any publication using information obtained from this study, and my confidentiality as a participant in this study will remain secure. | □ | □ |  |
| 1. Data collected for this study become property of Maastricht University and will not be shared with external parties. Personal information collected about me that can identify me, such as my name or age will not be shared beyond the primary researcher. | □ | □ |  |
| 1. Data (transcripts and recordings) will be stored with care and for 10 years after publication, as per compliance with Maastricht University’s Research Data Management Code of Conduct. | □ | □ |  |
| 1. The data will be stored on the researcher’s secure laptop during the thesis period (May-August 2024) and on Maastricht University’s data storage facilities later. I understand that this is a secure location. | □ | □ |  |
| I give my full consent for participation in this study.  **Signatures**  **Participant**  _____________ _____________________ ________  Name of participant Signature Date and location |  |  |  |
|  |  |  |  |

**Researcher**

I did my best to ensure that the participant understands to what they are freely consenting, I gave the participant the opportunity to ask questions and I confirm that the participant has given consent freely.

________________________ __________________ ________

Researcher name Signature Date and location
