# Supplementary material for: Barriers to leprosy elimination in Bolivia: Exploring perspectives and experiences of medical professionals and leprosy patients–A phenomenological study
Source: PLoS Negl Trop Dis. 2025 Aug 11;19(8):e0013345. doi: 10.1371/journal.pntd.0013345 (PMC12338824; doi:10.1371/journal.pntd.0013345)
Supplement: S2 File — (DOCX) [file pntd.0013345.s002.docx]

Appendix 2 - Interview guides for Leprosy patients, Spanish and English

| Percepciones y experiencias con la lepra | 1. ¿Me puede explicar qué ha ocurrido desde el primer momento que encontraste una manchita, o se encontró mal, hasta que vino a Jorochito por primera vez? 2. ¿Me puede explicar qué ha ocurrido desde el momento que empezó el tratamiento hasta ahora? ¿Ha tenido alguna dificultad? 3. ¿Usted ha tenido alguna otra experiencia, momento o dificultad relacionado con la lepra que quiere compartir conmigo? |
| --- | --- |
| Experiencias con la búsqueda activa de casos | 1. ¿Le han visitado los doctores a su casa? ¿Cómo fue la visita? 2. ¿Ha tenido alguna dificultad en relación con la visita? |
| Recomendaciones para mejorar | 1. Desde su punto de vista, ¿que se tiene que hacer para mejorar la experiencia de los pacientes de lepra y para reducir los casos en Bolivia? |

| Perceptions and experiences with leprosy | 1. Can you explain to me what has happened since the first moment you noticed a mark on your skin, or you felt unwell, until you came to Jorochito for the first time? 2. Can you explain to me what has happened since you started treatment for leprosy until now? Have you experienced any difficulties? 3. Are there any other experiences, moments or difficulties that you have encountered due to your leprosy that you would like to share with me? |
| --- | --- |
| Experiences with ACF | 1. Have the doctors visited you in your household yet? If so, how did the visit go? 2. Did you encounter any difficulties associated with the visit? |
| Recommendations for improvement | 1. From your point of view, what should be done in order to improve the experiences of leprosy patients and ensure that there are less leprosy cases in Bolivia? |
