# Supplementary material for: Barriers to leprosy elimination in Bolivia: Exploring perspectives and experiences of medical professionals and leprosy patients–A phenomenological study
Source: PLoS Negl Trop Dis. 2025 Aug 11;19(8):e0013345. doi: 10.1371/journal.pntd.0013345 (PMC12338824; doi:10.1371/journal.pntd.0013345)
Supplement: S3 File — (DOCX) [file pntd.0013345.s003.docx]

Appendix 3 - Interview guides for medical professionals, Spanish and English

##

| Percepciones y experiencias con el control de la lepra | 1. ¿Usted piensa que existen dificultades con respecto al control de la lepra en Bolivia? ¿Cuáles? 2. ¿Usted se enfrentó o se enfrenta con dificultades durante su trabajo como Doctora con respecto a la lepra? 3. Desde su punto de vista, con qué dificultades se enfrentan las pacientes para alcanzar tratamiento, ¿diagnosis de la lepra y completar el tratamiento? 4. ¿Existen otros obstáculos para el control de la lepra o la eliminación de la lepra en Bolivia de que aún no hemos hablado? |
| --- | --- |
| Papel de la búsqueda activa de casos | 1. Desde su punto de vista, ¿qué papel tiene la búsqueda activa de casos para el control de la lepra? ¿Es importante? |
| Recomendaciones para mejorar el control de la lepra | 1. ¿Que se tiene que hacer para alcanzar el control de la lepra y prevenir casos nuevos? |

##

| Perceptions and experiences with leprosy control | 1. Do you think there are difficulties with leprosy control in Bolivia? What are they? 2. Have you faced or are you facing difficulties in your work as a doctor with regard to leprosy? 3. From your point of view, what difficulties do patients face in getting treatment, being diagnosed with leprosy and completing their treatment? 4. Are there other obstacles to leprosy control or leprosy elimination in Bolivia that we have not yet discussed? |
| --- | --- |
| Role of ACF | 1. From your point of view, what role does active case finding play in leprosy control? Is it important? |
| Recommendations for improvement | 1. What needs to be done to achieve leprosy control and prevent new cases? |
