# Supplementary material for: Barriers to leprosy elimination in Bolivia: Exploring perspectives and experiences of medical professionals and leprosy patients–A phenomenological study
Source: PLoS Negl Trop Dis. 2025 Aug 11;19(8):e0013345. doi: 10.1371/journal.pntd.0013345 (PMC12338824; doi:10.1371/journal.pntd.0013345)
Supplement: S4 File — (DOCX) [file pntd.0013345.s004.docx]

**Appendix 4 - Participant information sheets, Leprosy patients and medical professionals, Spanish and English**

Ficha informativa para la participación de pacientes con lepra en el estudio

“Obstáculos para la eliminación de la lepra en Bolivia”

Me llamo Paula Messa-Carmona y soy una estudiante que está realizando una investigación sobre la lepra en Bolivia. Me gustaría invitarle a participar en mi estudio. Antes de decidir si desea participar, lea con atención esta ficha de información y póngase en contacto conmigo si desea más información. Mis datos de contacto se encuentran más abajo.

Detalles del estudio

Este estudio forma parte de mi máster en Salud Global, en la Universidad de Maastricht, Países Bajos. El estudio cuenta con el apoyo de la Asociación Alemana de Lucha contra la Lepra (DAHW) y la Fundación Damien, ambas comprometidas en la eliminación de la lepra en Bolivia. Con su apoyo, el estudio también se publicará en una publicación científica. Este estudio pretende explorar las barreras para la eliminación de la lepra en Bolivia, desde la perspectiva de los pacientes de lepra y de los médicos. También pretende explorar el valor de la búsqueda activa de casos para la eliminación de la lepra. Se desea que los resultados del estudio contribuyan a la futura eliminación de la lepra en Bolivia, aportando su contribución a las políticas sanitarias y a las intervenciones de salud.

Su participación

Este estudio requiere su participación, como persona con diagnóstico confirmado de lepra. Para participar debe ser mayor de 18 años, tener un diagnóstico confirmado de lepra, estar recibiendo o haber recibido tratamiento para la lepra y poder hablar español. Para participar debe haber leído y comprendido esta información sobre el estudio y sus derechos como participante, desear tomar parte en este estudio y dar su consentimiento para su participación. Si no puede dar su consentimiento para participar o los médicos consideran que se encuentra demasiado mal para participar en el estudio, su participación no será possible.

La participación en el estudio incluye una entrevista, que se espera que dure entre 30 y 45 minutos. Esta entrevista se realizará en persona en su hospital y en español. Se le harán algunas preguntas sobre su experiencia con la lepra en Bolivia y sobre cualquier problema al que se haya enfrentado en relación con el diagnóstico, el tratamiento o la prevención de la lepra. Usted no tiene que discutir nada que no se sientan cómodos haciéndolo.

Derechos de los participantes

Su participación en este estudio es confidencial y su identidad permanecerá anónima. Sólo yo, como investigadora, conoceré su nombre, edad y lugar de residencia. Una vez procesadas las entrevistas, su identidad quedará anonimizada, tanto para el resto de mi estudio como en la publicación científica.

Su participación en el estudio es totalmente voluntaria. Si considera que se le ha informado suficientemente sobre el estudio y desea participar, se le invitará a firmar un documento de consentimiento.

Tiene derecho a hacer una pausa durante la entrevista si así lo desea. También tiene derecho a parar la entrevista en cualquier momento y a retirarse del estudio si así lo desea, sin necesidad de indicar el motivo.

Grabación de audio

La participación en el estudio incluye la grabación de audio de las entrevistas. Debe dar su consentimiento a la grabación de las entrevistas para poder participar.

Uso, archivo y manejo de datos

Los datos de las entrevistas se utilizarán para escribir una tesis para la finalización del máster en la Universidad de Maastricht, y para una publicación en una publicación científica con DAHW. Los datos se archivarán durante 10 años tras la publicación del artículo en una publicación científica, de acuerdo con el Código de Conducta de la Universidad de Maastricht. Los datos archivados serán anónimos. Los datos se archivarán en primer lugar en el ordenador portátil protegido con contraseña de la investigadora, en una carpeta protegida con contraseña, y posteriormente en las infraestructuras de archivo de datos de la Universidad de Maastricht.

Posibles riesgos y mitigación

No está obligado a participar en el estudio. La participación requiere su total consentimiento. Tiene derecho a hacer cualquier pregunta sobre el estudio y su participación en el estudio, y a que se respondan sus preguntas antes de consentir su participación.

No debería sentirse obligado a participar en este estudio. Su participación en el estudio es independiente de los hospitales, los médicos y la atención que recibe, por lo que su participación no tendrá ningún efecto sobre ellos. No se le recompensará ni remunerará por su participación en el estudio, pero se le reembolsarán los gastos de viaje si corresponde.

Debe estar consciente de que pueden surgir sentimientos de malestar durante la entrevista, debido a la discusión de experiencias pasadas con la lepra. Soy consciente del estigma asociado a la lepra y soy sensible a ello. Habrá opciones de apoyo para usted si lo necesita durante la entrevista. Tiene derecho a pausar la entrevista en cualquier momento, puede tomarse un descanso durante la entrevista si lo desea, o puede parar la entrevista y retirarse del estudio sin dar ninguna explicación.

¿Preguntas?

Debe estar suficientemente informado sobre el estudio para participar y dar su consentimiento informado. Si tiene alguna pregunta sobre el estudio, póngase en contacto conmigo. Mis datos están a continuación.

Información de contacto

Nombre: Paula Messa-Carmona

Email: [p.messacarmona@student.maastrichtuniversity.nl](mailto:p.messacarmona@student.maastrichtuniversity.nl)

Número de teléfono: +44 7436832932

Information sheet for the participation of leprosy patients in the study

“Barriers to leprosy elimination in Bolivia”

Study details

This study is a part of my masters in Global Health, at Maastricht University, The Netherlands. The study is supported by the German Leprosy Relief Association (DAHW) and the Damien Foundation, both of which have been involved in leprosy elimination in Bolivia. With their support, the study will also be published in a scientific journal. This study aims to explore the barriers to leprosy elimination in Bolivia, from the perspective of leprosy patients and health professionals. It also aims to explore the role of active case finding for leprosy elimination. It is hoped that the results of the study will contribute to the future elimination of leprosy in Bolivia, by contributing to health policies or health interventions.

Participant involvement

This study requires the participation of leprosy patients and health professionals working to diagnose and treat leprosy in Bolivia. To participate, leprosy patients must be aged 18 or over, must have a confirmed leprosy diagnosis, must be undergoing treatment for leprosy and must be able to speak Spanish. To participate, leprosy patients must give full informed consent. If leprosy patients are unable to give informed consent or are considered to be too unwell to participate in the study by doctors, participation in the study will not be possible.

Participation in the study includes an interview, which is expected to last 30-45 minutes. This interview will be conducted in Spanish, and participants will be asked a few questions regarding their experience with leprosy in Bolivia and their perspective on the barriers to leprosy elimination. Participants do not have to discuss anything they do not feel comfortable doing so.

Participant rights

Participation in the study is fully voluntary. If you feel you have been sufficiently informed about the study and you would like to participate, you will be invited to sign a consent form.

Participants have the right to take a break during the interview if they wish. Participants also have the right to stop the interview at any time and withdraw from the study if they wish, without providing a reason.

Participants have the right to remain anonymous in the results of the study, the full write up of the study and the publication in the journal. Participants can also chose to not be anonymous, and for their participation in the study to be credited. The preference of participants must be discussed with the researcher. If participants wish to remain anonymous, only the researcher will know their full name. Once the interview is transcribed into text, the names of participants will be anonymised, and this will continue throughout the rest of the study.

Audio recording

Participation in the study involves the recording of interviews. Participants must consent to the recording of interviews to be able to participate.

Use, storage, and management of data

Data from the interviews will be used to write a thesis for the completion of the masters at Maastricht University, and for a publication in a scientific journal with DAHW. Data will be stored for 10 years after the publication of the article in a scientific journal, as per Maastricht University’s Code of Conduct. The stored data will include participant anonymisation, if applicable. Data will be stored in secure online locations, including the researcher’s password protected laptop in a password protected folder, and on Maastricht University’s data storage facilities.

Possible risks and mitigation

Participants are not obliged to participate in the study. Participation requires the full informed consent of participants. Participants have the right to ask any questions about the study and their involvement in the study, and to have their questions answered before consenting to participation.

Participants should not feel a sense of obligation to participate in the study. Participation in the study is independent from the hospitals, doctors, and care of the participants, so participation will have no effect on these. Participants will also not be rewarded or remunerated for their participation in the study.

Participants should be aware that feelings of distress may arise during the interview, due to discussing past, negative experiences with leprosy. The researcher is aware of the stigma associated with leprosy and is sensitive to this. There are support options for participants if required during the interview. Participants have the right to pause the interview at any time, can take a break away from the interview if desired, or can stop the interview and withdraw from the study without providing a reason.

Questions?

Participants must be sufficiently informed about the study to participate and give informed consent. If participants have any questions about the study, they can discuss these with the researcher.

Contact information

Name: Paula Messa-Carmona

Email: [p.messacarmona@student.maastrichtuniversity.nl](mailto:p.messacarmona@student.maastrichtuniversity.nl)

Phone number: +44 7436832932

Ficha informativa para la participación de médicos en el estudio

“Obstáculos para la eliminación de la lepra en Bolivia”

Me llamo Paula Messa-Carmona y soy una estudiante que está realizando una investigación sobre la lepra en Bolivia. Me gustaría invitarle a participar en mi estudio. Antes de decidir si desea participar, lea con atención esta ficha de información y póngase en contacto conmigo si desea más información. Mis datos de contacto se encuentran más abajo.

Detalles del estudio

Este estudio forma parte de mi máster en Salud Global, en la Universidad de Maastricht, Países Bajos. El estudio cuenta con el apoyo de la Asociación Alemana de Lucha contra la Lepra (DAHW) y la Fundación Damien, ambas comprometidas en la eliminación de la lepra en Bolivia. Con su apoyo, el estudio también se publicará en una publicación científica. Este estudio pretende explorar las barreras para la eliminación de la lepra en Bolivia, desde la perspectiva de los pacientes de lepra y de los médicos. También pretende explorar el valor de la búsqueda activa de casos para la eliminación de la lepra. Se desea que los resultados del estudio contribuyan a la futura eliminación de la lepra en Bolivia, aportando su contribución a las políticas sanitarias y a las intervenciones de salud.

Su participación

Este estudio requiere su participación, como médico que trabaja en el diagnóstico, tratamiento y control de la lepra en Bolivia. Para participar, debe ser mayor de 18 años, médico con experiencia en el control de la lepra y con pacientes con lepra y debe hablar español. Para participar debe haber leído y comprendido esta información sobre el estudio y sus derechos como participante, desear tomar parte en este estudio y dar su consentimiento para su participación. Si no puede dar su consentimiento informado, su participación no será possible.

La participación en el estudio incluye una entrevista, que se espera que dure entre 30 y 45 minutos. Esta entrevista se realizará en persona en su hospital y en español. Se le harán algunas preguntas sobre su experiencia con la lepra en Bolivia y sobre cualquier problema al que se haya enfrentado en relación con la atención de la lepra, durante su función como médico pero también en su vida personal. No es necesario que hable de nada con lo que no se sienta cómodo.

Derechos de los participantes

Su participación en este estudio es confidencial y su identidad permanecerá anónima. Sólo yo, como investigadora, conoceré su nombre, edad y lugar de residencia. Una vez procesadas las entrevistas, su identidad quedará anonimizada, tanto para el resto de mi estudio como en la publicación científica.

Su participación en el estudio es totalmente voluntaria. Si considera que se le ha informado suficientemente sobre el estudio y desea participar, se le invitará a firmar un documento de consentimiento.

Tiene derecho a hacer una pausa durante la entrevista si así lo desea. También tiene derecho a parar la entrevista en cualquier momento y a retirarse del estudio si así lo desea, sin necesidad de indicar el motivo.

Grabación de audio

La participación en el estudio incluye la grabación de audio de las entrevistas. Debe dar su consentimiento a la grabación de las entrevistas para poder participar.

Uso, archivo y manejo de datos

Los datos de las entrevistas se utilizarán para escribir una tesis para la finalización del máster en la Universidad de Maastricht, y para una publicación en una publicación científica con DAHW. Los datos se archivarán durante 10 años tras la publicación del artículo en una publicación científica, de acuerdo con el Código de Conducta de la Universidad de Maastricht. Los datos archivados serán anónimos. Los datos se archivarán en primer lugar en el ordenador portátil protegido con contraseña de la investigadora, en una carpeta protegida con contraseña, y posteriormente en las infraestructuras de archivo de datos de la Universidad de Maastricht.

Posibles riesgos y mitigación

No está obligado a participar en el estudio. La participación requiere su total consentimiento. Tiene derecho a hacer cualquier pregunta sobre el estudio y su participación en el estudio, y a que se respondan sus preguntas antes de consentir su participación.

No debería sentirse obligado a participar en este estudio. Su participación en el estudio es independiente de los hospitales, los médicos y la atención que recibe, por lo que su participación no tendrá ningún efecto sobre ellos. No se le recompensará ni remunerará por su participación en el estudio, pero se le reembolsarán los gastos de viaje si corresponde.

Debe estar consciente de que pueden surgir sentimientos de malestar durante la entrevista, debido a la discusión de experiencias pasadas con la lepra. Soy consciente del estigma asociado a la lepra y soy sensible a ello. Habrá opciones de apoyo para usted si lo necesita durante la entrevista. Tiene derecho a pausar la entrevista en cualquier momento, puede tomarse un descanso durante la entrevista si lo desea, o puede parar la entrevista y retirarse del estudio sin dar ninguna explicación.

¿Preguntas?

Debe estar suficientemente informado sobre el estudio para participar y dar su consentimiento informado. Si tiene alguna pregunta sobre el estudio, póngase en contacto conmigo. Mis datos están a continuación.

Información de contacto

Nombre: Paula Messa-Carmona

Email: [p.messacarmona@student.maastrichtuniversity.nl](mailto:p.messacarmona@student.maastrichtuniversity.nl)

Número de teléfono: +44 7436832932

Information sheet for the participation of medical professionals in the study

“Barriers to leprosy elimination in Bolivia”

Study details

This study is a part of my masters in Global Health, at Maastricht University, The Netherlands. The study is supported by the German Leprosy Relief Association (DAHW) and the Damien Foundation, both of which have been involved in leprosy elimination in Bolivia. With their support, the study will also be published in a scientific journal. This study aims to explore the barriers to leprosy elimination in Bolivia, from the perspective of leprosy patients and health professionals. It also aims to explore the role of active case finding for leprosy elimination. It is hoped that the results of the study will contribute to the future elimination of leprosy in Bolivia, by contributing to health policies or health interventions.

Participant involvement

This study requires the participation of leprosy patients and health professionals working to diagnose and treat leprosy in Bolivia. To participate, health professionals must be aged 18 or over, must be medical doctors with experience in leprosy control and with leprosy patients and must speak Spanish. To participate, health professionals must give full informed consent. If health professionals are unable to give informed consent, participation in the study will not be possible.

Participation in the study includes an interview, which is expected to last 30-45 minutes. This interview will be conducted in Spanish, and participants will be asked a few questions regarding their experience with leprosy in Bolivia and their perspective on the barriers to leprosy elimination. Participants do not have to discuss anything they do not feel comfortable doing so.

Participant rights

Participation in the study is fully voluntary. If you feel you have been sufficiently informed about the study and you would like to participate, you will be invited to sign a consent form.

Participants have the right to take a break during the interview if they wish. Participants also have the right to stop the interview at any time and withdraw from the study if they wish, without providing a reason.

Participants have the right to remain anonymous in the results of the study, the full write up of the study and the publication in the journal. Participants can also chose to not be anonymous, and for their participation in the study to be credited. The preference of participants must be discussed with the researcher. If participants wish to remain anonymous, only the researcher will know their full name. Once the interview is transcribed into text, the names of participants will be anonymised, and this will continue throughout the rest of the study.

Audio recording

Participation in the study involves the recording of interviews. Participants must consent to the recording of interviews to be able to participate.

Use, storage, and management of data

Data from the interviews will be used to write a thesis for the completion of the masters at Maastricht University, and for a publication in a scientific journal with DAHW. Data will be stored for 10 years after the publication of the article in a scientific journal, as per Maastricht University’s Code of Conduct. The stored data will include participant anonymisation, if applicable. Data will be stored in secure online locations, including the researcher’s password protected laptop in a password protected folder, and on Maastricht University’s data storage facilities.

Possible risks and mitigation

Participants are not obliged to participate in the study. Participation requires the full informed consent of participants. Participants have the right to ask any questions about the study and their involvement in the study, and to have their questions answered before consenting to participation.

Participants should not feel a sense of obligation to participate in the study. Participation in the study is independent from participants’ roles as medical professionals, and participation will not affect any relationship with other doctors or patients. Participation in the study will also not affect any support or relationship with the DAHW or the Damien Foundation. Participants will also not be rewarded or remunerated for their participation in the study.

Participants should be aware that feelings of distress may arise during the interview, due to discussing past, negative experiences with leprosy. The researcher is aware of the stigma associated with leprosy and is sensitive to this. There are support options for participants if required during the interview. Participants have the right to pause the interview at any time, can take a break away from the interview if desired, or can stop the interview and withdraw from the study without providing a reason.

Questions?

Participants must be sufficiently informed about the study to participate and give informed consent. If participants have any questions about the study, they can discuss these with the researcher.

Contact information

Name: Paula Messa-Carmona

Email: [p.messacarmona@student.maastrichtuniversity.nl](mailto:p.messacarmona@student.maastrichtuniversity.nl)

Phone number: +44 7436832932
