# Supplementary material for: Barriers to leprosy elimination in Bolivia: Exploring perspectives and experiences of medical professionals and leprosy patients–A phenomenological study
Source: PLoS Negl Trop Dis. 2025 Aug 11;19(8):e0013345. doi: 10.1371/journal.pntd.0013345 (PMC12338824; doi:10.1371/journal.pntd.0013345)
Supplement: S1 Data — (DOCX) [file pntd.0013345.s005.docx]

Appendix 5 - abstract in spanish

Abstracto

Contexto: La eliminación de la lepra ha vuelto a entrar recientemente en la esfera sanitaria global, con la estrategia de la Organización Mundial de la Salud (OMS) «Hacia la lepra cero» (2021-2030). Anteriormente, su eliminación se había definido como una prevalencia de menos de 1 caso por cada 10.000, que se alcanzó a nivel mundial en el año 2000, lo que condujo a una gran reducción de los recursos dedicados al control de la lepra y a su abandono a escala global y nacional. A pesar de ello, la lepra siguió proliferando y afectando a cientos de miles de personas cada año.

Métodos: El estudio explora las barreras a la eliminación de la lepra en Bolivia, utilizando

un estudio fenomenológico, para descubrir las percepciones y experiencias de los pacientes de lepra y de los profesionales médicos con respecto a la lepra en Bolivia. También explora el papel de la búsqueda activa de casos para la eliminación de la lepra en Bolivia. Se hicieron entrevistas semiestructuradas en profundidad en español, principalmente en el hospital dermatológico Jorochito, el centro nacional de referencia para la lepra en Bolivia.

Resultados: Las barreras para la eliminación de la lepra en Bolivia están presentes a nivel de proveedores, pacientes, gobierno, sociedad y comunidad. Entre ellas se encuentran la escasa financiación de la sanidad, la falta de formación del personal, la escasa adherencia al tratamiento, la organización centralizada del diagnóstico y el tratamiento de la lepra y el bajo nivel de conocimiento en temas de salud.

Conclusiones: Las barreras para la eliminación de la lepra en Bolivia son complejas, están interconectadas y ancladas en la sociedad boliviana. Se debe dar prioridad a la eliminación de la lepra a escala global y nacional, para aumentar la financiación y la importancia, continuar la búsqueda activa de casos y promover soluciones nacionales para el control sostenible de la lepra.
